# Carbon nanotubes and nanobelts as potential materials for biosensor

**DOI:** 10.1038/s41598-023-29862-9

**Published:** 2023-02-22

**Authors:** Seyyed Mostafa Monavari, Farah Marsusi, Nafiseh Memarian, Mohammad Qasemnazhand

**Affiliations:** 1 Faculty of Physics, Semnan University, P.O. Box 35195-363, Semnan, Iran; 2 Department of Physics and Energy Engineering, Amirkabir University of Technology, P.O. Box 15875-4413, Tehran, Iran

**Keywords:** Biomaterials, Environmental biotechnology, Nanobiotechnology

## Abstract

We investigate the electronic response of single-walled carbon nanotubes (SWCNTs) and a carbon nanobelt (CNB) to N-linked and O-linked SARS-CoV-2 spike glycoproteins, using ab initio quantum mechanical approach. The CNTs are selected from three zigzag, armchair, and chiral groups. We examine the effect of carbon nanotube (CNT) chirality on the interaction between CNTs and glycoproteins. Results indicate that the chiral semiconductor CNTs clearly response to the presence of the glycoproteins by changing the electronic band gaps and electron density of states (DOS). Since the changes in the CNTs band gaps in the presence of N-linked are about two times larger than the changes in the presence of the O-linked glycoprotein, chiral CNT may distinguish different types of the glycoproteins. The same results are obtained from CNBs. Thereby, we predict CNBs and chiral CNTs have suitable potential in sequential analysis of N- and O-linked glycosylation of the spike protein.

## Introduction

The outbreak of the SARS-CoV-2 pandemic (COVID-19) reveals the urgent need for high-accurate, sensitive, selective, and inexpensive long-term biomaterial detection techniques^1^. One of the great challenges in the detection of SARS-CoV-2 is that it damages the target RNA when opening its viral capsid. Therefore, small pieces of RNA are release into the bloodstream, which makes it hard to detect by traditional sensors^1^. Biosensors are exceptional alternatives to traditional detecting tools^2^. Nanotechnology opens new windows in developing and designing biosensors in combination with nanomaterials^3^. Among nanomaterials, carbon nanotubes (CNTs) are hollow, one-dimensional (1D) carbon fibers. CNTs are longer than a micrometer and have a diameter ranging between 1 and 20 nm^4^. CNTs show a unique combination of electrical, optical, mechanical, and chemical properties^5,6^. Consequently, CNTs offer great promises for a variety of applications, including biosensing^7^. When combined with specific polymers, CNTs can be used as optical sensors for a wide range of analytes, including reactive oxygen species, insulin, dopamine, and nitric oxide^8^. The emission wavelengths of single-walled CNTs (SWCNTs) are close to the infrared range^9^, in which water and blood have limited interference^10^. CNT-based sensors react to the specific analyte, and this reaction manifests in the intensity of the fluorescence and causes red or blue shifts in wavelengths^11^. Due to a very large specific surface area^12^, CNTs can combine with many glycoprotein molecules^7,13^. This property makes CNTs a suitable candidate to detect SARS-CoV2 virus^7,13,14^.

SARS-CoV-2 spike is characterized by a large number of N-linked and O-linked glycosylation proteins^15,16^. Glycosylation of spike virus protein plays a vital role in virus stability and its ability in suppressing immunological surveillance^17^. Hence, it is important to characterize the glycosylation sequence. There are two main strategies in employing a biosensor^18^: (i) detection of viral nucleic acid sequence, (ii) detection of viral biomolecules, such as surface proteins. Determining the sequence of N-linked or O-linked glycan proteins can lead to detection of both spike mutation sequence and surface protein.

The present study aimed to investigate and analyze the ability of CNTs and carbon nanobelts (CNBs) in detection and quantification of N-linked and O-linked sequences. Our study is based on the changes in the electronic properties of CNTs and CNBs in the presence of N-linked or O-linked glycan molecules, using density functional theory (DFT). CNBs are observed in SWCNTs laser vaporization process^19,20^. Over the last few years, there has been significant progress in the synthesis of carbon nanorings and CNBs^21^. CNTs with a selective and predictable chirality are synthesized using a bottom-up approach from a CNB template that corresponds to the target CNT structure^22^. Armchair CNBs (6,6), (8,8), (12,12) and more recently zigzag CNB(18,0), which have been considered the most difficult type to synthesize, among those have been successfully fabricated^21,23,24^.

In practice, depending on the manufacturing approach, CNTs can be implemented in the sensor platform in different ways. Owing to their electrical and electrochemical properties, CNTs are very promising materials for integration into electrochemical biosensors^7^. Recently, a biosensor for SARS-CoV-2 spike antigen detection, based on a CNT-field effect transistor has been developed which shows very sensitive, selective performance^25,26^ . CNTs are used as signal transducers in field-effect biosensors. Our results show that in addition to a signal transducer, CNT is capable of selective recognition of glycoproteins for virus detection.

**Figure 1:**
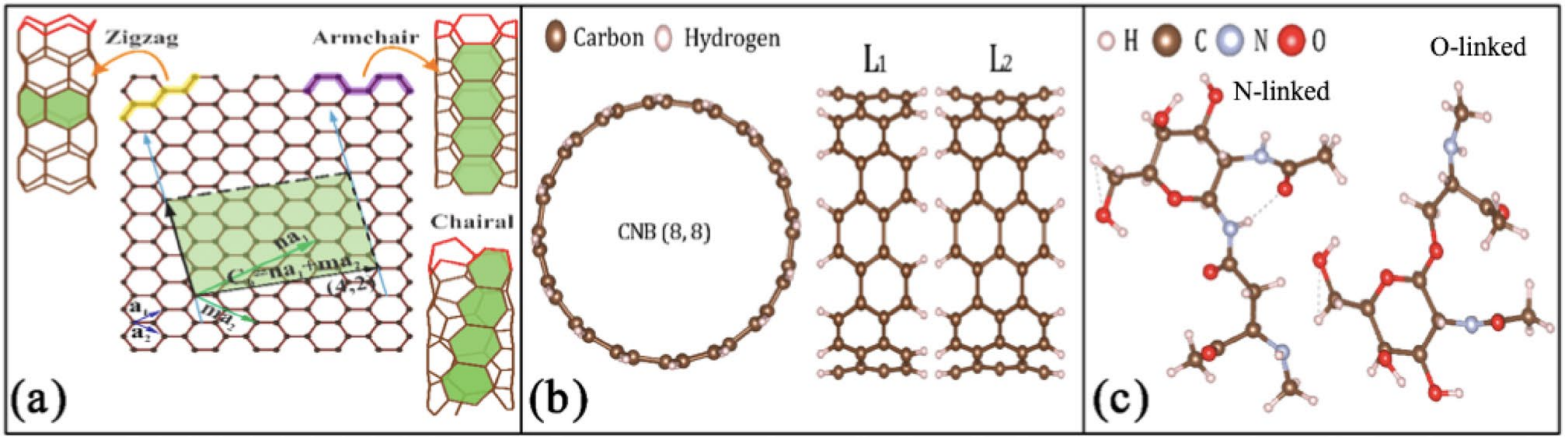
(**a**) The structure of SWCNT can be shown by a graphene sheet wrapped along a chiral vector **C**_h_. **a**_1_ and **a**_2_ show the graphene primitive vectors. Three types of CNT (zigzag, armchair, and chiral) are illustrated. (**b**) Cross-section and lateral surface of CNB(8,8) with Lengths L_1_ = 5.57 Å and L_2_ = 6.82 Å. (**c**) The simplest N- and O-linked of glycan molecular structures found in SARS-Cov-2.

Since electronic properties of CNTs highly depend on the chirality and structural geometry (i.e., whether they are semiconductors or metals)^27,28^ , we have built three groups of zigzag (*m* = 0), armchair (*n* = *m*), and general chiral (*n* > *m* > 0) CNTs, in size that allows computational capabilities. Here *n*, *m* are chiral indexes, as shown in Fig. 1a. We analyzed the variation in the electronic band structure and electron density of state (DOS) upon glycan adsorption. These properties characterize the electronic response of sensors^29^. Our results show that chiral semiconductor CNTs have the potential to be developed as viral sensing materials due to their high electrical response to virus glycoproteins. To investigate the capability of CNBs as biosensors, we have calculated the electronic gap, binding energy, chemical potential, ionization potential, electron affinity, chemical hardness, and electron felicity in the presence of a glycoprotein molecule. We also used time-dependent density functional theory (TD-DFT) to calculate the absorption spectra of a CNB sample and monitored the change in its spectrum after being introduced to the N-linked and O-linked glycan. Our results show that the presence of the N-linked or O-linked glycan molecules, can change the electronic band gap of the chiral semiconductor CNTs and CNBs, and therefore, they can be used for monitoring Corona-like virus spikes. Outcomes enable us to predict those structures that may show more sensitive optical and electronic responses in sensing devices, while our results can be detected experimentally through signals given by the built-in electronic device in biosensors.

## Results

### Symmetry-dependent electronic properties of CNT

The chirality (*n*,*m*) indicates how a graphene sheet has been folded to form a CNT. We can show the structure of an SWCNT by taking two hexagons of a graphene sheet and rolling up the sheet to overlap these two hexagons^30^. A vector that connects the center of two hexagons is named a chiral vector, as seen in Fig. 1a. Chiral vector **C**_*h*_ is written in terms of the primitive vectors **a**_1_ and **a**_2_ of the graphene lattice in the form of **C**_*h*_ = *n***a**_1_ + *m***a**_2_, with *n* and *m* being integer numbers. If *n* = *m* = *j*, with *j* being zero or any positive integer number, the resulting nanotube is called an armchair (*n*,*n*) CNT. A zigzag CNT(*n*,0) is formed when *n* = *j* and *m* = 0. Other combinations of *n* and *m* are called chiral CNT^31^. An SWCNT has metallic properties with a zero electronic gap, if *n* − *m* = 3*j*^32^, otherwise, the SWCNT behaves as a semiconductor with an electronic gap that depends on the chirality (*n*,*m*) and inversely proportions with CNT diameter^31^. Therefore, the geometry of a CNT plays an important role in its electronic properties. In the reciprocal space, the allowed states are called cutting lines. The chirality of the CNT determines the orientation of the cutting lines^33^. Depending on whether the cutting line crosses the degenerate **K** and **K**’ points of the Brillouin zone (BZ), an SWCNT behaves as either metallic (M) with *n* − *m* = 3*j*, or a semiconductor (S) with *n* − *m* ≠ 3*j*^34^. In a metallic CNT, the cutting lines crosse the **K** points. Depending on at what ratio the **K** points divide the cutting lines, the metallic CNTs can be classified into M1 or M2 types. Following Ref.^34^, we name the greatest common divisor of the (2*n* + *m*,2* m* + *n*) by d*R* and of the two integers (*n*,*m*) by d. In the M1-type CNT d*R* = d, while in the M2-type CNT d*R* = 3d^34^. On the other hand, CNT cutting lines do not crosse the **K** point in a semiconductor CNT. There are two types of semiconductor CNTs. If *n* − *m* = 3*j* + 1, the CNT is S1-type semiconductor and if *n* − *m* = 3*j* − 1, it is called S2-type CNT. In an S1-type CNT the distance of the **K** point from the nearest cutting line passing inside the BZ is two-thirds of the distance between two adjacent cutting lines, while for an S1-type, this is a one-third^34^. An armchair CNT (*n*,*n*) is always M2-type metallic. For a zigzag CNT three types are possible: M1-type (3*j*, 0), S1-type (3*j* + 1,0) or S2-type (3*j* + 2,0). For a chiral CNT each of the four types M1, M2, S1 (*n* − *m* = 3*j* + 1), and S2 (*n* − *m* = 3*j* + 2) is possible^34^.

### Pristine CNTs

**Figure 2:**
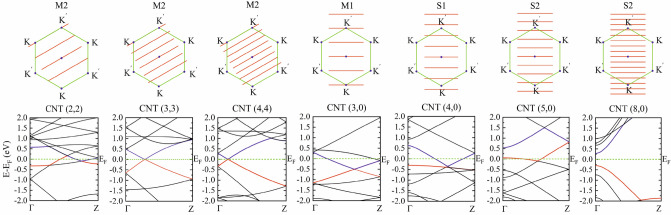
Top: cutting lines in zone folding illustration of armchair and zigzag CNTs. Bottom: the corresponding DFT electronic band structures. Valence and conduction bands are represented in red and blue colours, respectively. Fermi energy E_F_ is set to zero.

From the armchair group, we investigate the electronic band structure and electron DOS of the three (2,2), (3,3) and (4,4) CNTs. Here *n* − *m* = 0, and therefore, these CNTs should be metals, as confirmed also by the computational outcomes given in Table 1, Figs. 2 and S1 in supplementary data. The bands are extended from point Γ at the center of BZ to the boundary point at Z = π/c, where “c” shows the periodicity length along the tube axis. The corresponding cutting lines of these three CNTs cross both **K** and **K**’ points, which means they are M2-metallic. In the same way, the corresponding DFT-band structures indicate metallic features, with valence bands crossing the Fermi level.

We study four zigzag CNTs with chirality (3, 0), (4, 0), (5, 0) and (8, 0). The related data are given in Table 1. According to the *n* − *m* = 3*j* rule, CNT(3, 0) should be a metal, as it is truly predicted, and shown in Fig. 2, by cutting lines crossing the **K** or **K**’ points. Therefore, CNT(3,0) is an M1-metal type. Likewise, both the zone folding picture and the DFT band structure, shown in Fig. 2, predict CNT(8, 0) is a semiconductor. There is an exception for CNTs(4,0) and (5,0). Cutting line analysis of the zone folding approach in Fig. 2 predicts that these CNTs are semiconductor types S1 and S2, respectively. However, the corresponding DFT band structures in this figure and DOS presented in Figure S1 predict metallic features, despite *n* − *m* not being a multiple of 3. The same DFT predictions are previously reported^35,36^. In fact, the high curvatures of the wall structures in these two small tubes induce σ-π surface hybridization. Consequently, the relaxed structures show a significant deviation from the ideal rolled-up graphene sheet configuration, which leads to failure of the zone folding approach prediction. Previously, it was shown that zone folding approach prediction is reliable only for CNTs with diameters larger than 1 nm^37,38^.

**Figure 3:**
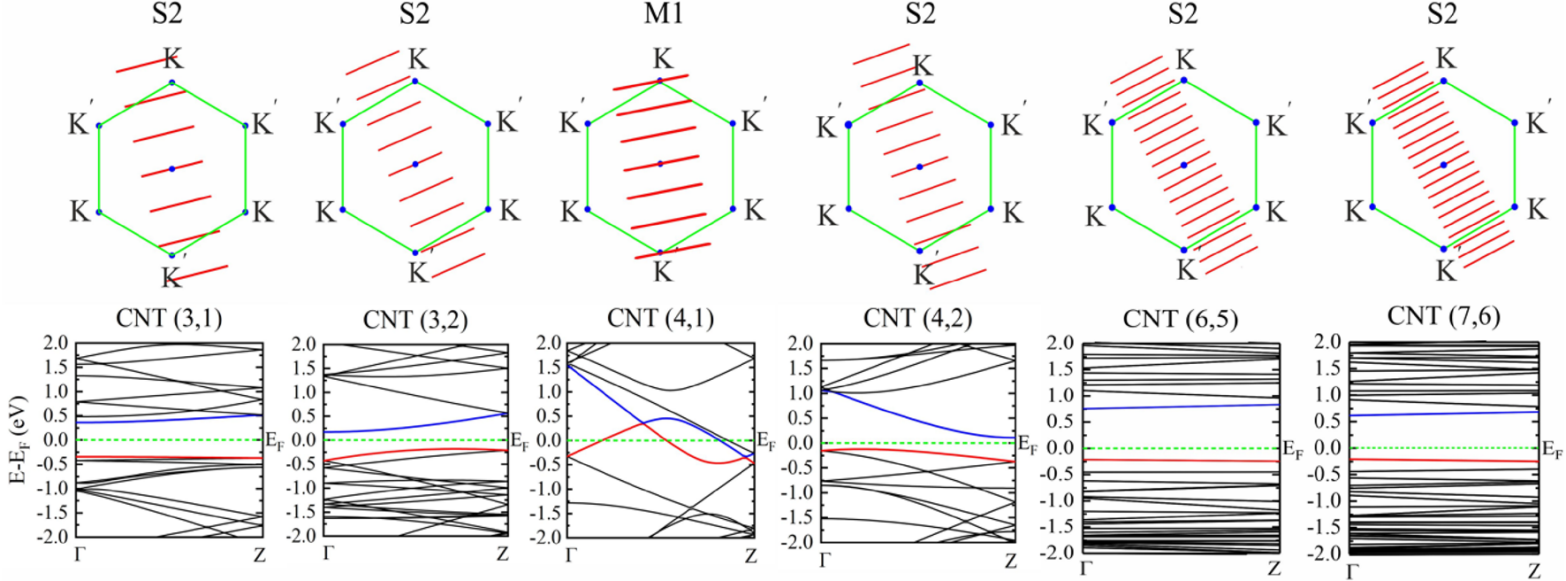
Top: cutting lines in zone folding illustration of chiral CNTs. Bottom: the corresponding DFT electronic band structures. Valence and conduction bands are represented in red and blue colours, respectively. Fermi energy E_F_ is set to zero.

Band structures and cutting lines in BZ of pristine chiral CNTs (3,1), (3,2), (4,1), (4,2), (6,5), and (7,6) are shown in Fig. 3 and the related data are given in Table 1. Among them, CNT(4,1) is a metal and the other chiral CNTs are semiconductors. Electronic states of CNT(4,1) cross **K** and **K**’ points separately, and therefore this nanotube is an M1-metallic. In the remaining chiral CNTs no electronic state crosses point **K**, or **K**’, and depending on the values of the *n* − *m*, they are S1- or S2-type semiconductors.

**Table 1:** Chirality (*n*,*m*),electronic feature (metal: M, semiconductor: S), type, and electronic bandgap (Egap) of pristine CNTs. Band gaps are compared with the previous data.

Chirality	Feature	Diameter (Å)	Number of atoms	CNT type	E_gap_ (eV)	Prev. work( eV)
(2,2)	M2	2.91	24	Armchair	0.0	0.0^39^
(3, 3)	M2	4.39	36	Armchair	0.0	0.0^40^
(4, 4)	M2	5.53	48	Armchair	0.0	0.0^39^
(3,0)	M1	2.69	24	Zigzag	0.0	0.0^39^
(4,0)	M1	3.37	32	Zigzag	0.0	0.0^39^
(5,0)	M1	4.10	40	Zigzag	0.0	0.0^39^
(8,0)	S2	6.36	32	Zigzag	0.598	0.56(LDA)^41^
						0.57(PBE)^42^
(3,1)	S2	3.97	52	Chiral	0.705	0.72(PBE)^39^
(3,2)	S1	3.60	76	Chiral	0.360	0.30(PBE)^39^
(4,1)	M1	3.74	28	Chiral	0.0	0.0^39^
(4,2)	S2	4.28	56	Chiral	0.233	0.19(PBE)^39^
(6,5)	S1	7.57	364	Chiral	0.967	1.272(Exp.)^43^
(7,6)	S1	8.83	508	Chiral	0.821	1.105(Exp.)^43^

### Pristine CNBs

Among synthesized CNB series, we investigate the electronic properties of CNB(8,8). To investigate the effect of the CNB length on the electronic properties, we added an additional layer to the initial L_1_ size CNB(8,8), see Fig. 1. By this, the length of the CNB increases from L_1_ = 5.57 Å to the L_2_ = 6.82 Å, and the optimized structures have the diameters of 1.15 Å and 1.16 Å, respectively. Figure 1c shows the optimized structures of these two CNBs. We have used DFT to predict several electrochemical indicators, including electronic gap (*E*_*gap*_*)*, chemical potential (μ), chemical hardness (η), electron affinity (*EA*), ionization potential (*IP*), binding energy (Δ*Ε*_Β_) and electron felicity (ω) to distinguish chemical features of the corresponding CNB + glycan compounds. These properties are defined as^44–46^:

$$EA = { } - E{_\text{LUMO}},{ }IP = { } - E{_\text{HOMO}},{ } E_{gap} = E_{LUMO} - E_{HOMO} ,$$

1 $$\mu = - \frac{IP + EA}{2} = - \frac{1}{2}\left( {E_{HOMO} + E_{LUMO} } \right),$$

$$\eta = \frac{1}{2}\left( {IP - EA} \right) = \frac{{\left[ {E_{LUMO} - E_{HOMO} } \right]}}{2}, \omega = \frac{{\mu^{2} }}{\eta },$$

$$\Delta E_{B} = \left[ {E\left( {glycan} \right) + E\left( {CNB} \right)} \right] - E\left( {glycan + CNB} \right),$$

## Discussion

**Figure 4:**
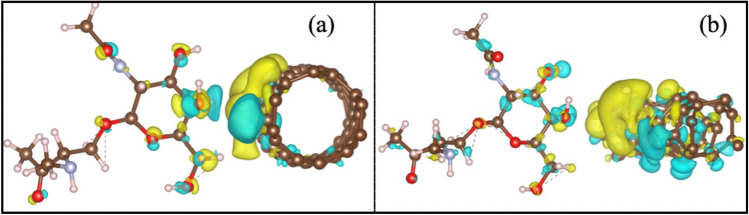
Δρ for (**a**): CNT(4,2) in the presence of an O-linked glycan molecule, (**b**): CNT(3,1) in the presence of an O-linked glycan molecule. Charge accumulation in yellow colour and charge depletion area in blue colour are shown.

CNT (CNB) and glycan molecules form a weak bound state through a van der Waals (vdW) interaction characterized by the charge density rearrangement at the compound interface. The corresponding calculated binding energies are about 0.3 eV with equilibrium distances larger than 3 Å. Charge density difference (Δρ) illustrates the change in the charge distribution in a compound relative to its isolated components. Here, Δρ is defined by ρ(CNT + glycan compound)-[ρ(isolated CNT) + ρ(isolated glycan)]. Δρ for CNT(3,1) and CNT(4,2) in the presence of glycan molecule are shown in Fig. 4. Figure confirms that after interaction no electric charge is shared between two species, but the charge density of both CNT and glycan molecule is perturbed in the interface. Formation of interfacial electric dipole and quadrupole moments is the consequent of charge accumulation (Δρ > 0 in yellow colour) and charge depletion (Δρ < 0 in blue colour) on interfacial surface.

**Figure 5:**
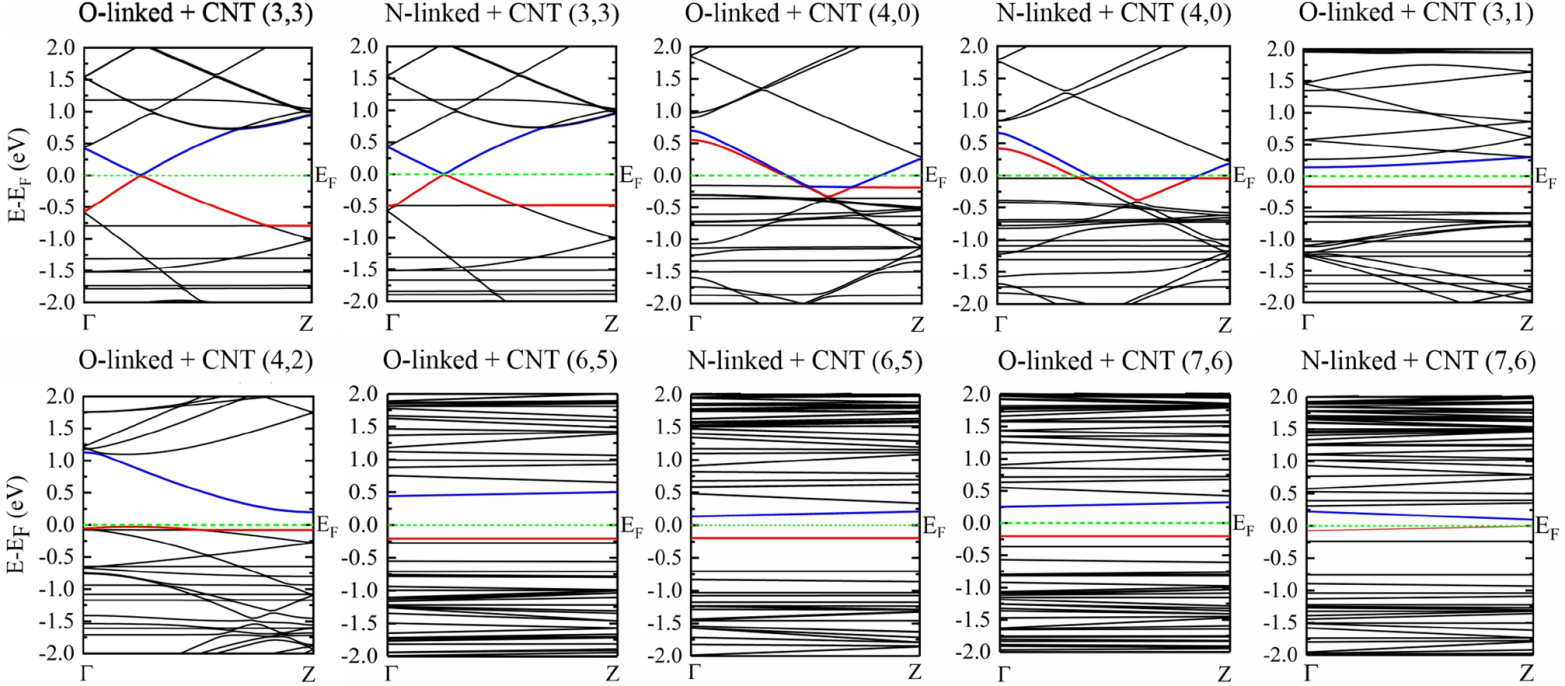
Band structure of semiconductor CNTs in the presence of glycan molecules. Valence and conduction bands are represented in red and blue colours, respectively. Fermi energy E_F_ is set to zero.

According to our outcomes, the metallic CNTs almost show no response to the presence of glycoproteins. However, the band structures and gaps of semiconductor CNTs are influenced by the glycoprotein molecules. Variation in the band gaps in the presence of the glycans respect to the pristine CNTs (∆Egap = Egap(CNT)-Egap(CNT + glycan)) are reported in Table 2. Data in Table 2 indicate that the electronic response of a semiconductor CNT to the presence of N-linked glycan is about two times larger than to O-linked glycan. Comparing the band structures of pristine CNTs with those of the CNT + N-linked complexes shows that both valence and conduction bands of CNT + N-linked complexes move toward the Fermi level, see Figs. 5 and S2.

**Table 2:** Number of atoms in the simulation super cell for CNT + glycan complexes, the corresponding electronic band gaps, Egap, in eV, and the variation in band gap, ∆Egap, compared to pristine CNT.

Complex	Number of atoms	E_gap_	ΔE_gap_
O-linked + CNT(3,1)	98	0.303	0.402
O-linked + CNT(4,2)	102	0.227	0.006
N-linked + CNT(6,5)	413	0.332	0.635
O-linked + CNT(6,5)	410	0.647	0.320
N-linked + CNT(7,6)	557	0.164	0.657
O-linked + CNT(7,6)	554	0.454	0.367

However, in interaction with O-linked glycan, this is the conduction band that mostly influenced by the interaction and moves toward Fermi level. As the valence band approaches the Fermi level, it is possible that the semiconductor tube becomes a metal, as seen for CNT(7,6) + N-linked complex, in which the valence band nearly coincides the Fermi level at the BZ boundary point, however it is still a semiconductor.

Now we discuss how CNB electrochemical features are influenced by the interaction with the glycan molecules. CNT(8,8) is an armchair 1D tube, which according to *m* − *n* = 3*j* law should be a metal with a zero band gap. In contrast, due to quantum confinement effect, zero-dimensional (0D) L_1_ size CNB(8,8) has relatively a wide electronic gap of 2.39 eV. With increasing the length of the CNB from L_1_ to L_2_, the 0D structure becomes closer to 1D CNT(8,8) and the gap reduces to 0.72 eV. Likewise, the electronic response of the CNB to the presence of the glycans decreases with increasing the length of the CNB, as one concludes from comparing the ∆Egap given in Table 3. According to the information given in Table 3, the CNB with length L_1_ is a better option for detecting glycans than CNB with longer length L_2_, owing to a better sensitivity, showing larger band gap variation, and more stability (higher chemical hardness). In addition, the binding energy for L_1_ size CNB + glycan complexes is larger than for L_2_ complexes.

**Table 3:** HOMO and LUMO level energies, HOMO-LOMO gap (Egap), change in HOMO–LUMO gap in the presence of glycan molecules (∆*E*gap) respect to pristine CNB, chemical potential (μ), ionization potential (IP), electron affinity (EA), chemical hardness (η), and electron felicity (ω). All quantities are given for both pristine CNB and CNB in the presence of N-linked and O-linked glycans. ∆*E*_B_ shows binding energy of glycan molecules to CNBs. All quantities are given in eV.

Structure	HOMO	LUMO	E_gap_	ΔE_gap_	μ	IP	EA	η	ω	ΔE_B_
N-linked glycan	− 4.48	− 1.56	2.92	–	3.02	4.48	1.56	1.46	3.12	–
O-linked glycan	− 4.90	− 2.08	2.82	–	3.49	4.90	2.08	1.41	4.32	–
CNB_1_ (8,8)	− 4.93	− 2.54	2.39	–	3.73	4.93	2.54	1.19	5.83	–
CNB_1_ (8,8) + N-linked	− 4.37	− 2.80	1.57	0.82	3.58	4.37	2.80	0.78	8.20	0.32
CNB_1_ (8,8) + O-linked	− 4.65	− 2.69	1.97	0.42	3.67	4.65	2.69	0.98	6.85	0.12
CNB_1_(8,8) + N- and O-linked	− 4.40	− 2.90	1.50	0.89	3.65	4.40	2.90	0.75	8.88	0.60
CNB_2_ (8,8)	− 4.06	− 3.35	0.72	–	3.71	4.06	3.35	0.36	19.18	–
CNB_2_ (8,8) + N-linked	− 4.32	− 3.62	0.70	0.02	3.97	4.32	3.62	0.35	22.56	0.30
CNB_2_ (8,8) + O-linked	− 4.18	− 3.49	0.70	0.02	3.84	4.18	3.49	0.35	21.10	0.13

**Figure 6:**
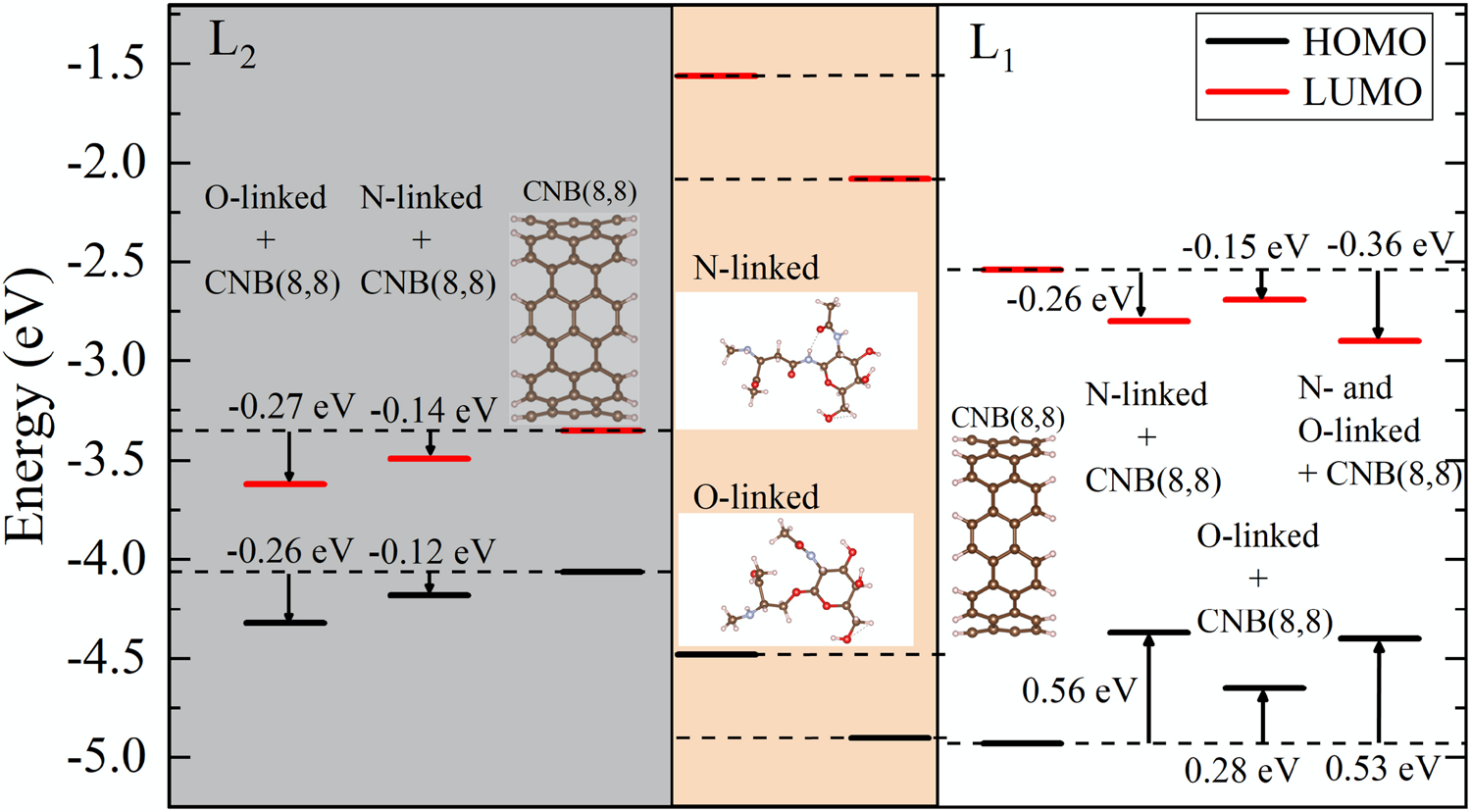
HOMO and LUMO energy levels of glycan molecules and pristine CNB(8,8) with lengths L_1_ and L_2_. Renormalized energy levels of CNBs in the presence of glycan molecules are shown.

In Fig. 6, we pictorially show the changes in the energy of HOMO and LUMO levels of CNBs in the presence of glycoprotein molecules. According to Fig. 6, at length L_1_, the presence of the glycan molecules pulls up the HOMO level and pushes down the LUMO level. Thus, the energy gap undergoes a serious change. However, at size L_2_, the glycan molecules reduce the energy of both HOMO and LUMO levels, and no significant change is achieved in the band gap.

Hydrogen bond strength could be classified into three groups depending on the amount of binding energy. Strong bond energies are greater than 0.65 eV, moderate bond energies happen at between 0.2 and 0.65 eV, and weak bonds energies are less than 0.2 eV^47^. The calculated binding energies given in Table 3, show that the hydrogen bonds strength for the CNB in the presence of the N-linked glycoprotein are moderate for both CNBs. While the corresponding values are weak for both sizes CNB in the presence of O-linked glycan. Also, binding energies given in Table 3 reveal that the vdW interactions between CNBs and glycan molecules are greater for smaller lengths.

**Figure 7:**
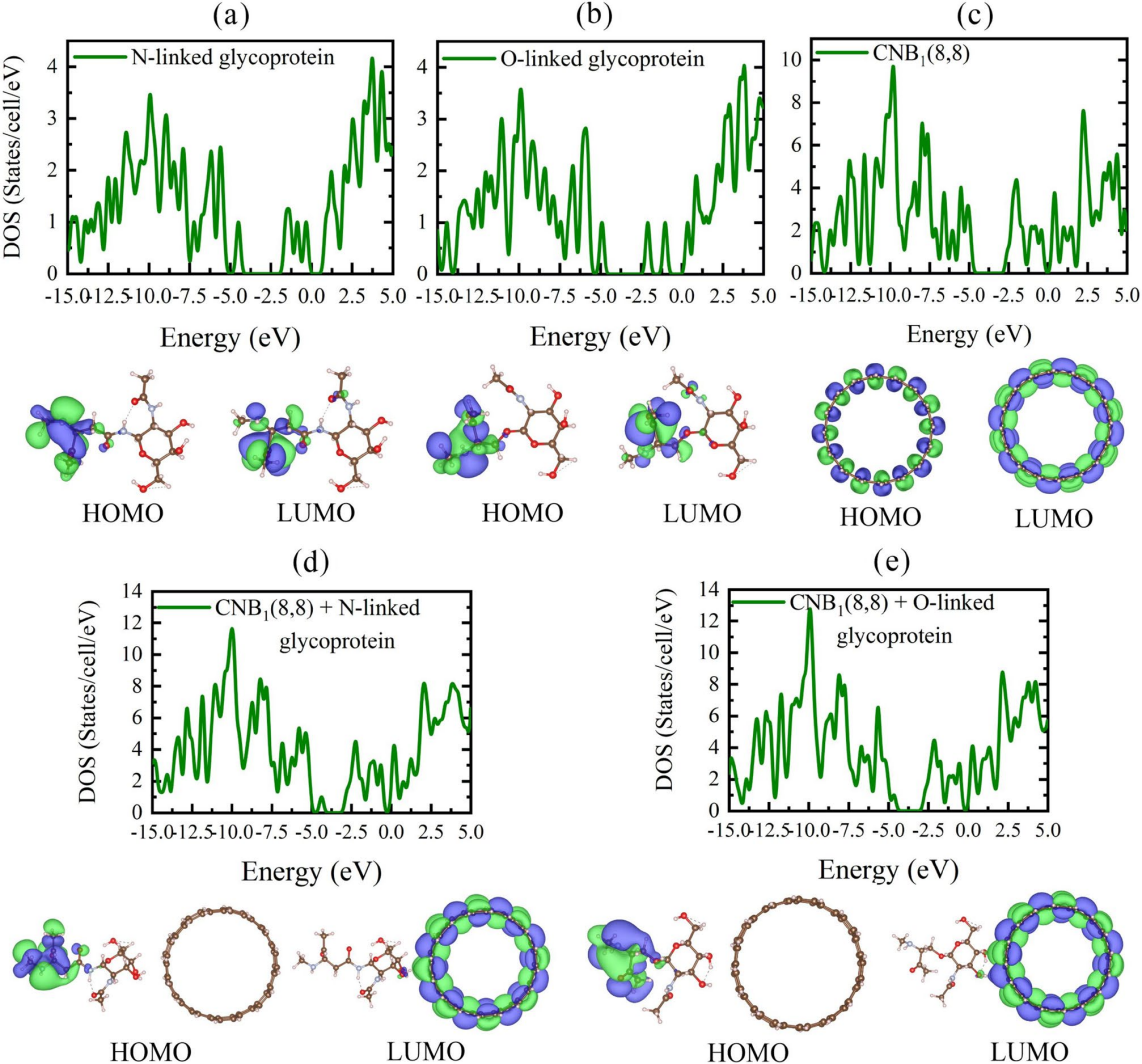
DOS, HOMO, and LUMO isosurface of (**a**): N-linked, (**b**): O-linked glycan molecules, (**c**): pristine CNB(8, 8), (**d**): CNB(8, 8) + N-linked, (**e**): CNB(8, 8) + O-linked glycan complexes.

The HOMO and LUMO orbitals representation of the individual glycans, L_1_ size CNB(8,8), and CNB(8,8) + glycan composite systems are shown in Fig. 7. Additional information is given in supplementary data. The introduction of high electronegativity anions by glycan molecules causes intense changes in the electronic properties of the CNB. The HOMO orbital isosurface shows a charge localization on the amine or ketone functional group of the glycan molecules, so that N-linked and O-linked groups suppress the electronic dynamics of the combined structure through nonbonding electron pairs in the functional groups. That means, the nonbonding pair of electrons in the nitrogen and oxygen atoms dominate the HOMO state and impose a new level inside the HOMO–LUMO gap of pristine CNB, as seen in Fig. 7. This finding is confirmed by the DOS diagrams given in Fig. 7. Also, the presence of the nitrogen and oxygen atoms increases the chemical potential of the L_1_ size CNB, and the electron can more easily be excited, or the structure can be ionized with less energy than before. As a result, changes in the electron DOS and the HOMO–LUMO orbital representation suggest the possibility of serious changes in the optical response of the CNB, due to interaction with the glycans. We will discuss this issue further.

**Figure 8:**
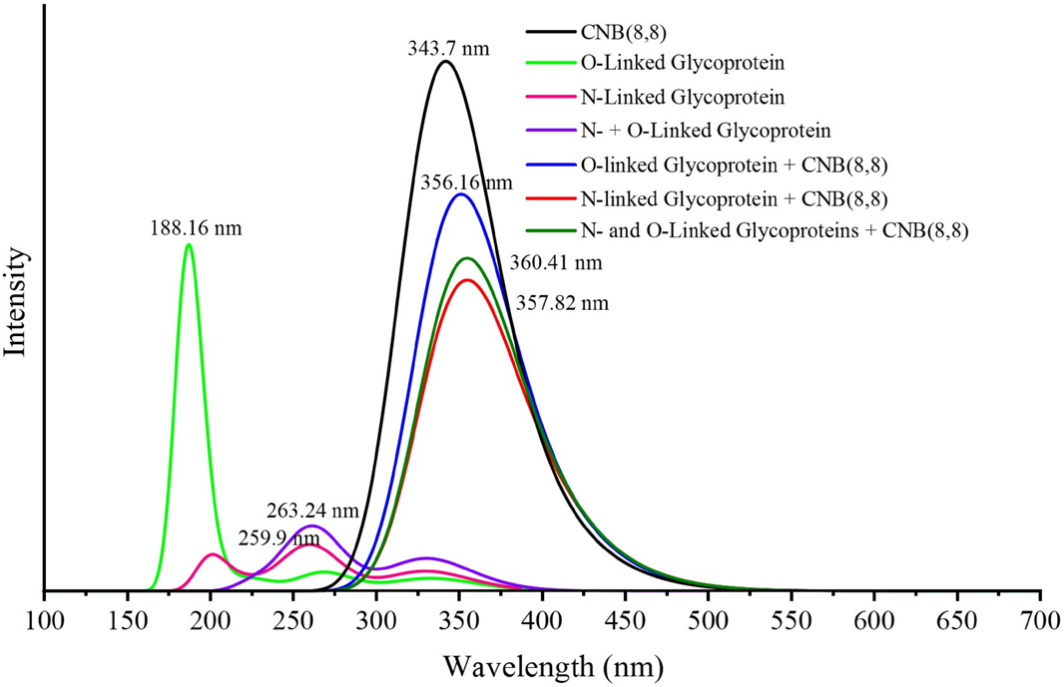
TD-B3LYP predicted absorption spectrum of pristine CNB, glycan molecules, and CNB + glycon complex. As the glycoprotein approaches the CNB, the absorption spectra shift toward the visible region.

The optical properties of sensor materials are of the important practical features of interest. Thereby, we show how the absorption spectrum of CNB is changed by the presence of the intended glycan. B3LYP functional takes advantage of the cancellation of localization and delocalization errors, which makes it a more reliable functional to predict absorption spectrum of materials^48,49^. Consequently, we use B3LYP in addition to the PBE functional in this section. TD-B3LYP predicted spectra of L_1_ size CNB in the presence of glycoprotein molecules are shown in Fig. 8. The corresponding TD-PBE spectra are given in Figure S3. B3LYP predicts that the glycan molecules have almost no absorption in the visible region. Similarly, the absorption spectrum of pristine CNB is almost in the ultraviolet area, and the absorption tails approach the visible region. By absorption of N-linked, O-linked, or simultaneously O- and N-linked glycans, the spectra of the CNB + glycan complexes show redshifts toward the visible region due to involvement in vdW interactions. Owing to stronger interaction, the absorption of N-linked glycan gives larger red shift than absorption of the O-linked glycan.

In conclusion, we investigated the change in the electronic properties of several CNTs and a selected CNB in the presence of N-linked and O-linked glycoprotein molecules. Our results show that, depending on the size and chirality, the semiconductor chiral CNTs can be good options to use as biosensor materials. Depending on the electronic gap and length of the nanobelt, CNB show appropriate electronic and optical sensitives that are required for biosensor materials. The present study gives important findings in diagnosis materials for viral diseases based on CNTs.

## Methods

Virus spikes are often decorated by a large number of N-linked and O-linked glycoproteins. The structures of the N-linked and O-linked glycans are taken from Ref.^50^ and shown in Fig. 1c. For smaller CNTs, calculations were performed within the framework of the plane-wave pseudopotential approach and Perdew-Burke-Ernzerhof (PBE) functional, using Quantum- Espresso computational package^51,52^.The Grimme’s vdW correction (PBE-D2) is added to our calculations to introduce the long-range effect of vdW interactions^53^. The simulation was performed at room temperature (300 K). The cutoff energy on the plane-wave kinetic energy was set to 45 Ha. A Monkhorst k-point mesh was used to sample the Brillouin zone^54^. The volume of the supercell and the atomic positions of the structures were optimized under conditions where the forces acting on atoms are less than 5 meV/Å. Periodic boundary conditions along the growth direction have been applied to all structures. Cutting lines are plotted using Wolfram Demonstrations project^33,55,56^.

Band structure of larger tubes CNTs(6,5) and (7,6), which have smaller wall curvatures, are calculated within self-consistent Slater Koster tight binding semi-empirical methods, as implemented in the DFTB + package, using fine BZ k-point mesh^57^. Previously, it was shown that semi-empirical tight binding method provides CNT band gap prediction in an accuracy comparable to DFT schemes^58^. For smaller CNT (3,1), we obtained a difference of about 0.1 eV in the band gap from PBE-DFT and DFTB. However, since our goal is to find the variation in band gap of CNT in the presence of glycan molecules, our results benefited from band gap cancelation errors. We applied PBE and hybrid B3LYP functionals and imported atomic orbitals using the LANL2DZ basis set to find the geometrical orientation and electronic properties of the N-linked and O-linked glycoprotein molecules near CNBs^59^. The UV–Vis spectra were calculated in the TD-DFT framework. All CNB calculations were performed using the Gaussian 98 package^60^.

## Supplementary Information

Supplementary Information.

## Data Availability

Data generated or analysed during this study are included in this published article and its supplementary information file.
