# Supplementary material for: Carbon nanotubes and nanobelts as potential materials for biosensor
Source: Sci Rep. 2023 Feb 22;13:3118. doi: 10.1038/s41598-023-29862-9 (PMC9945837; doi:10.1038/s41598-023-29862-9)
Supplement: Supplementary file 1 — Supplementary Information. [file 41598_2023_29862_MOESM1_ESM.docx]

**Carbon nanotubes and nanobelts as potential materials for viral biosensor**

**Seyyed Mostafa Monavari**1**, Farah Marsusi**2,***, Nafiseh Memarian**1,+**, and Mohammad Qasemnazhand**2

1Faculty of Physics, Semnan University, P.O. Box: 35195-363, Semnan, Iran

2Department of Physics and Energy Engineering, Amirkabir University of Technology, P.O. Box 15875-4413, Tehran, Iran
[*marsusi@aut.ac.ir](mailto:*marsusi@aut.ac.ir)

**Figure S1**. Density of states for selected CNTs presented in Table 1. Fermi energy is set to zero in each figure.


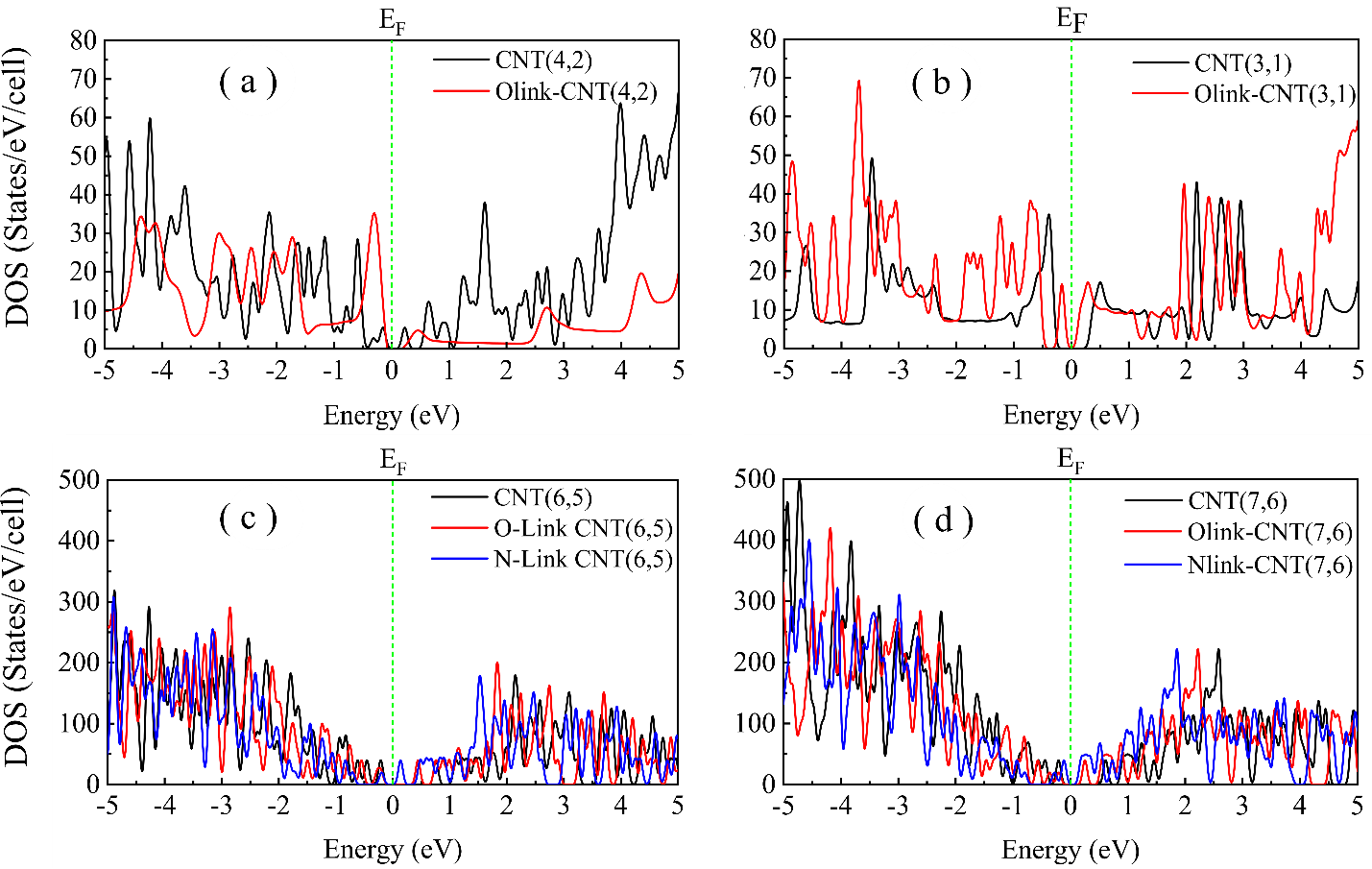


**Figure S2**. Density of states for studied chiral carbon nanotubes (pristine CNT and in the presence of glycan molecules). Fermi energy is set to zero in each figure.


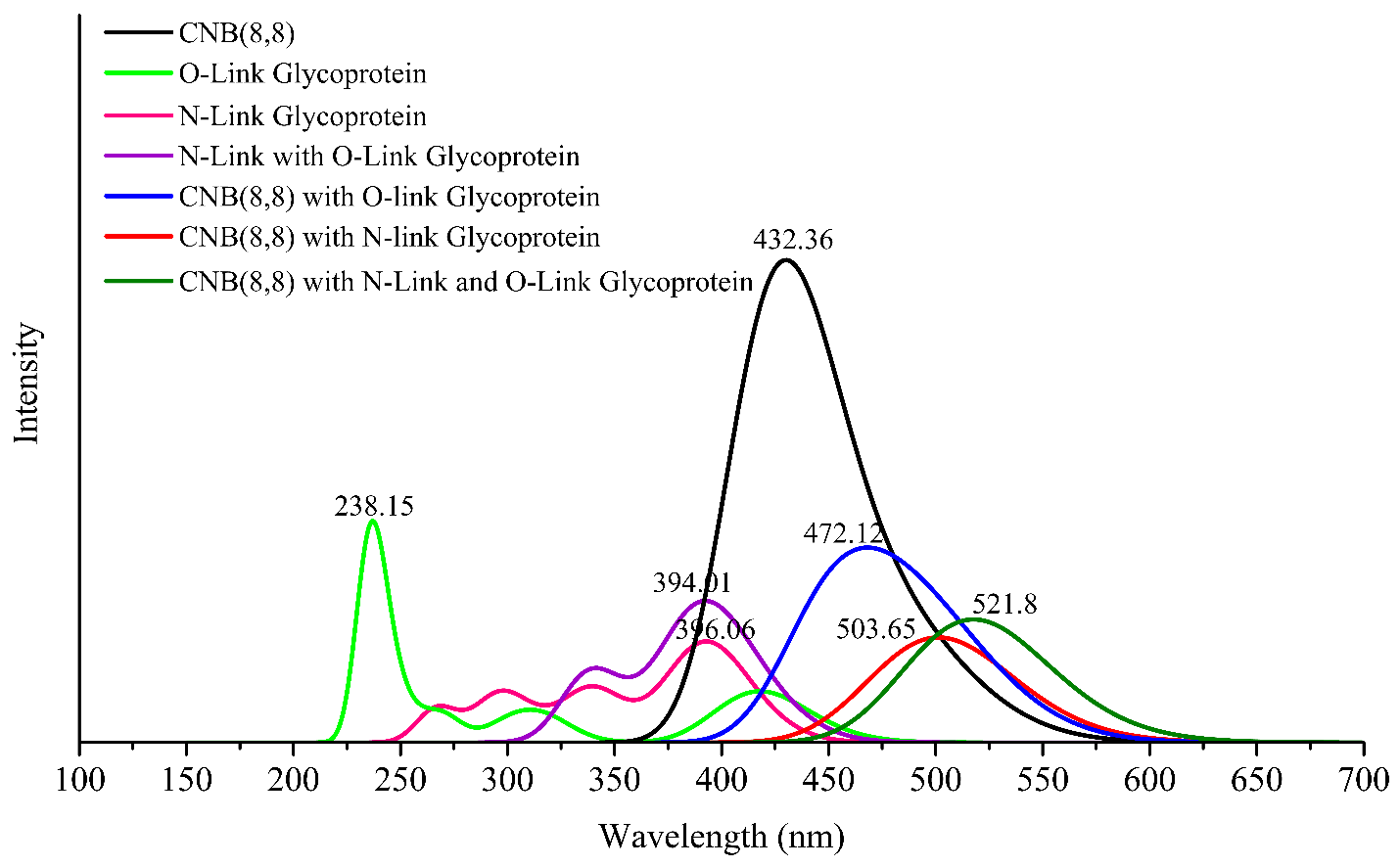


**Figure S3.** Variation of absorption spectra of L1 size CNB (8, 8) in the presence of glycan molecules, predicted by PBE TD-DFT.

**Pseudopotentials** **used for CNT calculations:**

C 12.01070 C.pbe-n-rrkjus_psl.1.0.0.UPF

H 1.00800 H.pbe-rrkjus_psl.1.0.0.UPF

N 14.00700 N.pbe-n-rrkjus_psl.1.0.0.UPF

O 15.99900 O.pbe-n-rrkjus_psl.1.0.0.UPF

**Monkhorst mesh**

K_POINTS crystal

50

0.0000000000 0.0000000000 0.0000000000

0.0000000000 0.0000000000 0.0102040816

0.0000000000 0.0000000000 0.0204081633

0.0000000000 0.0000000000 0.0306122449

0.0000000000 0.0000000000 0.0408163265

0.0000000000 0.0000000000 0.0510204082

0.0000000000 0.0000000000 0.0612244898

0.0000000000 0.0000000000 0.0714285714

0.0000000000 0.0000000000 0.0816326531

0.0000000000 0.0000000000 0.0918367347

0.0000000000 0.0000000000 0.1020408163

0.0000000000 0.0000000000 0.1122448980

0.0000000000 0.0000000000 0.1224489796

0.0000000000 0.0000000000 0.1326530612

0.0000000000 0.0000000000 0.1428571429

0.0000000000 0.0000000000 0.1530612245


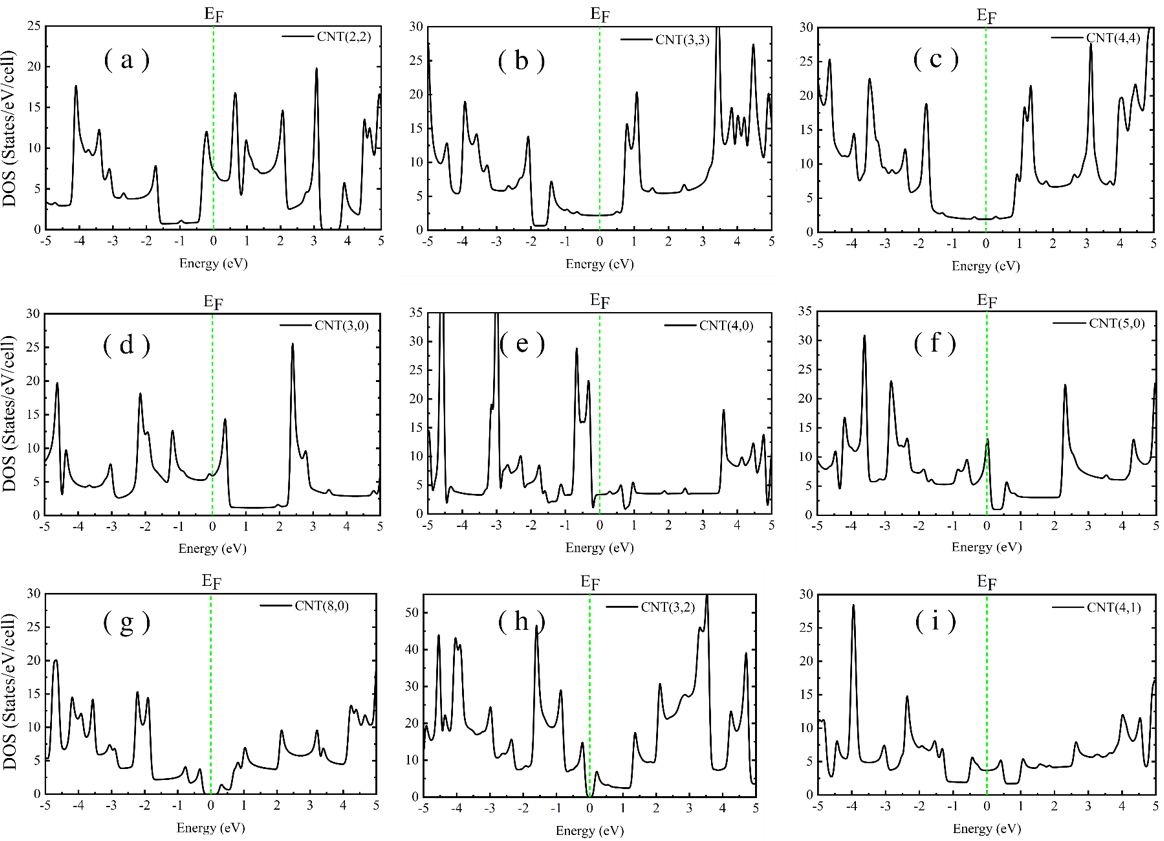
 0.0000000000 0.0000000000 0.1632653061

0.0000000000 0.0000000000 0.1734693878

0.0000000000 0.0000000000 0.1836734694

0.0000000000 0.0000000000 0.1938775510

0.0000000000 0.0000000000 0.2040816327

0.0000000000 0.0000000000 0.2142857143

0.0000000000 0.0000000000 0.2244897959

0.0000000000 0.0000000000 0.2346938776

0.0000000000 0.0000000000 0.2448979592

0.0000000000 0.0000000000 0.2551020408

0.0000000000 0.0000000000 0.2653061224

0.0000000000 0.0000000000 0.2755102041

0.0000000000 0.0000000000 0.2857142857

0.0000000000 0.0000000000 0.2959183673

0.0000000000 0.0000000000 0.3061224490

0.0000000000 0.0000000000 0.3163265306

0.0000000000 0.0000000000 0.3265306122

0.0000000000 0.0000000000 0.3367346939

0.0000000000 0.0000000000 0.3469387755

0.0000000000 0.0000000000 0.3571428571

0.0000000000 0.0000000000 0.3673469388

0.0000000000 0.0000000000 0.3775510204

0.0000000000 0.0000000000 0.3877551020

0.0000000000 0.0000000000 0.3979591837

0.0000000000 0.0000000000 0.4081632653

0.0000000000 0.0000000000 0.4183673469

0.0000000000 0.0000000000 0.4285714286

0.0000000000 0.0000000000 0.4387755102

0.0000000000 0.0000000000 0.4489795918

0.0000000000 0.0000000000 0.4591836735

0.0000000000 0.0000000000 0.4693877551

0.0000000000 0.0000000000 0.4795918367

0.0000000000 0.0000000000 0.4897959184

0.0000000000 0.0000000000 0.5000000000

**Coordinates of Armchair CNT (3,3)**

C 0.631149426 0.500027900 0.083330924

C 0.600487174 0.584282722 0.083330887

C 0.565547934 0.613595130 0.250000119

C 0.477248888 0.629169702 0.249999936

C 0.434398843 0.613571525 0.083331113

C 0.376759878 0.544885593 0.083330720

C 0.368852501 0.499972692 0.250000179

C 0.399514254 0.415718030 0.249999972

C 0.434451452 0.386403421 0.083330987

C 0.522750671 0.370828823 0.083330649

C 0.565599568 0.386429790 0.250000193

C 0.623238904 0.455115209 0.249999923

C 0.631149647 0.500027904 0.416669246

C 0.600487337 0.584282860 0.416669039

C 0.565548650 0.613596797 0.583330694

C 0.477249280 0.629171416 0.583330667

C 0.434398700 0.613571734 0.416669011

C 0.376759651 0.544885680 0.416669295

C 0.368850353 0.499972096 0.583330754

C 0.399512663 0.415717140 0.583330961

C 0.434451350 0.386403203 0.416669306

C 0.522750720 0.370828584 0.416669333

C 0.565601300 0.386428266 0.583330989

C 0.623240349 0.455114320 0.583330705

C 0.631147500 0.500027308 0.749999821

C 0.600485746 0.584281970 0.750000028

C 0.565548548 0.613596579 0.916669013

C 0.477249329 0.629171177 0.916669351

C 0.434400432 0.613570210 0.749999807

C 0.376761096 0.544884791 0.750000077

C 0.368850574 0.499972100 0.916669076

C 0.399512826 0.415717278 0.916669113

C 0.434452066 0.386404870 0.749999881

C 0.522751112 0.370830298 0.750000064

C 0.565601158 0.386428475 0.916668887

C 0.623240122 0.455114407 0.916669280

**Coordinates of CNT (4,0) next to N-link glycoprotein**

C 0.692668133 0.397442790 0.097710007

C 0.775611495 0.414960276 0.097307896

C 0.709096783 0.313338873 0.097274398

C 0.791824201 0.330908106 0.097609395

C 0.730340855 0.424256357 0.183065771

C 0.684435892 0.352177008 0.182705024

C 0.800205122 0.376073974 0.182676040

C 0.754310373 0.304160079 0.183071042

H 0.574806234 0.497567328 0.277874695

C 0.730485029 0.424257850 0.347302441

C 0.684525099 0.352059347 0.347672099

C 0.800269193 0.376039845 0.347718160

C 0.754346196 0.304103512 0.347321913

H 0.490542973 0.481485233 0.350985888

H 0.539550642 0.416392101 0.360549296

O 0.405336122 0.463146953 0.403289285

H 0.243376862 0.540838390 0.348522858

H 0.270023691 0.438773543 0.341125873

H 0.324708619 0.511531843 0.370862559

H 0.330035441 0.419433969 0.346327472

H 0.546581061 0.580451067 0.306073941

H 0.519296489 0.654717553 0.280386896

H 0.648869824 0.523585681 0.350442215

C 0.693112246 0.397165557 0.432611519

C 0.775810855 0.414970869 0.433048691

C 0.709128185 0.313128137 0.433108577

C 0.791922240 0.330821268 0.432803508

H 0.621394929 0.437046487 0.406914871

C 0.297620634 0.423388144 0.419050340

C 0.583330783 0.503788960 0.404549677

N 0.529531422 0.572699657 0.409284097

C 0.411551078 0.496058680 0.498663624

C 0.498143337 0.491270504 0.475931105

C 0.549611592 0.424314522 0.485157109

C 0.239195531 0.536994316 0.477742494

H 0.469103125 0.669534130 0.402815314

O 0.623418547 0.535682838 0.418269995

C 0.506098816 0.656598182 0.402895705

C 0.322433484 0.509777136 0.501059959

H 0.285872129 0.388903719 0.466500040

C 0.594394749 0.455375295 0.481780972

H 0.586397812 0.357809637 0.521323720

H 0.222349858 0.501897462 0.501364152

C 0.540255728 0.527056855 0.484345862

O 0.515286007 0.347629913 0.560471409

H 0.376224967 0.561951081 0.562573854

H 0.214783677 0.565015404 0.519364027

N 0.455923118 0.511211556 0.544356278

N 0.285779675 0.543295691 0.553220648

O 0.510896772 0.448933009 0.561194027

C 0.370445169 0.523235123 0.574518079

H 0.487961874 0.366683711 0.593857882

C 0.507784391 0.609209520 0.488551963

C 0.556295136 0.377465640 0.573905984

C 0.308808850 0.457867762 0.550152194

H 0.527669353 0.682515281 0.469543201

C 0.693188180 0.397148514 0.597622215

C 0.775780508 0.414924169 0.597410530

C 0.709121567 0.313204331 0.597401782

C 0.791924301 0.330804934 0.597543020

H 0.460025864 0.541164266 0.613113788

H 0.548213981 0.533398752 0.610599534

O 0.612183284 0.461375317 0.638763700

H 0.282739634 0.541563573 0.674111795

O 0.490065067 0.603932669 0.622508107

C 0.730474032 0.424206754 0.683054922

C 0.684458412 0.352134775 0.682675175

C 0.800303658 0.375983556 0.682655923

C 0.754362770 0.304066828 0.683084728

H 0.369548779 0.515020487 0.702551685

H 0.636449719 0.486358159 0.633650964

H 0.565067531 0.385401373 0.698823996

O 0.305938869 0.447950481 0.691270370

C 0.730336593 0.424199123 0.847261729

C 0.684417292 0.352213092 0.847626288

C 0.800243958 0.376025239 0.847684999

C 0.754331428 0.304113922 0.847248504

C 0.692744854 0.397400982 0.932770128

C 0.775588783 0.414922538 0.932970417

C 0.709095803 0.313371991 0.932948119

C 0.791829541 0.330893306 0.932773361

**Carbon nanobelt (CNB)**

**CNB (8,8) next to N-link glycoprotein**

# opt pbepbe/lanl2dz

**Coordinates of CNB (8,8) next to N-link glycoprotein**

C -1.19434300 2.45164600 -1.26081000

C -1.61020100 1.12993500 -1.32627500

C -1.55746400 0.28133700 -0.16120200

C -1.42803100 -1.16818400 -0.26727600

C -1.34233800 -1.83367800 -1.54381000

C -0.69540400 -3.05531200 -1.66388200

C -0.09145000 -3.69603800 -0.52198300

C 1.02948800 -4.61883000 -0.65990600

C 1.59573500 -4.94298600 -1.94523100

C 2.91888100 -5.34454500 -2.06141500

C 3.76882900 -5.45056500 -0.90176800

C 5.21838900 -5.31268300 -0.99371800

C 5.88122300 -5.06010500 -2.24861400

C 7.10984800 -4.41620800 -2.29144900

C 7.76313500 -3.97948500 -1.08291600

C 8.70318800 -2.86368200 -1.07537800

C 9.03230700 -2.13621300 -2.27580000

C 9.44974700 -0.81405000 -2.21831400

C 9.56717000 -0.12619600 -0.95665800

C 9.43499700 1.32316700 -0.85327700

C 9.17524500 2.14831800 -2.00658200

C 8.52889700 3.37006100 -1.88116000

C 8.09740000 3.85326100 -0.59327000

C 6.97631200 4.77644400 -0.45202700

C 6.23889200 5.25982800 -1.59260200

C 4.91617200 5.66192000 -1.47167200

C 4.23701500 5.60737900 -0.20139000

C 2.78798400 5.46603900 -0.10764300

C 1.95476700 5.37618100 -1.28068000

C 0.72640100 4.73212100 -1.23957300

C 0.24454900 4.13183600 -0.02088900

C -0.69490800 3.01672300 -0.03067300

C -0.96039900 2.30297200 1.18628600

C -1.40623800 0.91536500 1.11879100

C -1.47928800 0.07459100 2.29018900

C -1.35127500 -1.30779800 2.18788500

C -1.14735500 -1.94275200 0.90911100

C -0.46453200 -3.22456400 0.78155400

C 0.06480400 -3.92407500 1.92507700

C 1.13469500 -4.79819900 1.79320200

C 1.74803200 -5.03653900 0.51053900

C 3.13904400 -5.45943200 0.38807000

C 3.97875800 -5.66014200 1.54240200

C 5.35750000 -5.52889500 1.45476800

C 5.99438300 -5.18759200 0.20741700

C 7.28679500 -4.51193800 0.16207000

C 7.99895400 -4.14869900 1.36165800

C 8.89192000 -3.08647000 1.36896500

C 9.13614200 -2.31259300 0.17731500

C 9.57510300 -0.92255500 0.23766900

C 9.79000900 -0.24521800 1.49198500

C 9.66666500 1.13354000 1.59033500

C 9.31861300 1.93194300 0.44150000

C 8.64022600 3.21695300 0.57327800

C 8.28272400 3.76051500 1.85980300

C 7.21595600 4.63774800 1.99414300

C 6.43185000 5.03343900 0.85096900

C 5.04013000 5.45233900 0.97812200

C 4.37044600 5.48915900 2.25405500

C 2.99197000 5.35591600 2.34347700

C 2.18542300 5.17591700 1.16244100

C 0.89433700 4.49735400 1.20602700

C 0.35353000 3.96928300 2.43329600

C -0.54313900 2.90976700 2.42505700

H -0.85081600 2.47501300 3.38076000

H 0.72137200 4.33824700 3.39509600

H 2.53312700 5.29338200 3.33452400

H 4.95599000 5.52620200 3.17727400

H 6.91670400 4.95232700 2.99820000

H 8.79199400 3.40962300 2.76210300

H 9.74190600 1.59919800 2.57731400

H 9.95854900 -0.82447800 2.40444400

H 9.33846400 -2.78160000 2.31994400

H 7.76913200 -4.64891200 2.30693300

H 5.95118200 -5.59412300 2.37124300

H 3.52778000 -5.82506700 2.52530800

H 1.57120700 -5.23450600 2.69630600

H -0.30901800 -3.69779500 2.92804100

H -1.30600100 -1.90012700 3.10633800

H -1.52129800 0.52542500 3.28618800

H 0.16914300 4.60229700 -2.17175000

H 2.32742700 5.73464200 -2.24452000

H 4.36384000 5.93171800 -2.37643500

H 6.68867800 5.22428500 -2.58912200

H 8.26311900 3.91977100 -2.78878100

H 9.39938900 1.77288100 -3.00929000

H 9.59741900 -0.26794200 -3.15454800

H 8.86342100 -2.59238300 -3.25562500

H 7.53366900 -4.15687800 -3.26595500

H 5.37457100 -5.28892200 -3.19063200

H 3.33831000 -5.48876700 -3.06133300

H 1.01239800 -4.78293700 -2.85660300

H -0.56688100 -3.48412600 -2.66186300

H -1.70624700 -1.33470700 -2.44586200

H -1.90926200 0.71178400 -2.29120400

H -1.16401000 3.03834100 -2.18346100

C -7.95843300 -0.00023600 0.08827200

C -6.20025600 1.16781400 -1.16161800

C -5.08826900 0.33499800 -0.49695800

C -5.59888800 -0.27268300 0.83332400

C -6.92124100 -1.03881400 0.61534000

H -8.00228200 0.90318300 0.73527900

H -4.23375700 1.01109700 -0.27137900

H -5.76541800 0.55128000 1.56296300

H -6.78214400 -1.78433600 -0.19325000

H -6.37200500 2.07857300 -0.54243000

O -7.46951800 0.41551400 -1.25456600

C -5.88814100 1.59160700 -2.60168100

H -4.95825700 2.18465400 -2.63368600

H -5.76700300 0.68405500 -3.22347500

O -6.94027500 2.46893400 -3.11517100

H -7.80597500 2.00917400 -2.95522600

O -4.65418200 -0.72780500 -1.40270900

H -4.17303700 -1.40293100 -0.85183200

O -4.62945700 -1.24099700 1.37831400

H -3.79897700 -0.78319300 1.68256900

N -7.28370700 -1.73192400 1.86547800

H -6.59815200 -1.65577100 2.62192500

C -8.15938100 -2.79984300 1.89930400

O -8.88908700 -3.11699100 0.90073600

C -8.21493700 -3.58285600 3.20307800

H -9.25373400 -3.59421200 3.57436500

H -7.92845400 -4.63003100 3.00384100

H -7.55774300 -3.17449000 3.98934400

N -9.27755800 -0.54723500 -0.05084300

H -9.35939400 -1.57891800 -0.07117000

C -10.40118100 0.26434700 -0.02506600

O -10.32571700 1.52316300 0.14041300

C -11.72555500 -0.47875500 -0.19855400

H -11.80669800 -1.29582300 0.54344400

H -11.75115900 -0.94593900 -1.20397400

C -12.95273600 0.44327000 -0.04371100

H -12.86117600 1.02397400 0.89157500

N -14.16981400 -0.40103700 0.04545600

H -14.29529200 -0.92869000 -0.83156700

C -13.04091600 1.42596600 -1.25162800

C -13.04872200 2.91166100 -0.96592700

H -13.86183600 3.16270900 -0.25697000

H -12.09344100 3.16803100 -0.47134000

H -13.16895100 3.49337200 -1.89327100

O -13.14661900 0.94935300 -2.41703100

C -15.41006500 0.29632400 0.44850100

H -16.22182500 -0.44520100 0.55302900

H -15.25752700 0.76420000 1.43850100

H -15.76158900 1.08789800 -0.25453200
